# Barriers to Antiretroviral Therapy Adherence Among HIV-Positive Hispanic and Latino Men Who Have Sex with Men —United States, 2015–2019

**DOI:** 10.15585/mmwr.mm6940a1

**Published:** 2020-10-09

**Authors:** Stacy M. Crim, Yunfeng Tie, Linda Beer, John Weiser, Sharoda Dasgupta

**Affiliations:** 1 Division of HIV/AIDS Prevention, National Center for HIV/AIDS, Viral Hepatitis, STD, and TB Prevention, CDC.

During 2018, estimated incidence of human immunodeficiency virus (HIV) infection among Hispanic and Latino (Hispanic/Latino) persons in the United States was four times that of non-Hispanic White persons (*1*). Hispanic/Latino men who have sex with men (MSM) accounted for 24% (138,023) of U.S. MSM living with diagnosed HIV infection at the end of 2018 (*1*). Antiretroviral therapy (ART) adherence is crucial for viral suppression, which improves health outcomes and prevents HIV transmission (*2*). Barriers to ART adherence among Hispanic/Latino MSM have been explored in limited contexts (*3*); however, nationally representative analyses are lacking. The Medical Monitoring Project reports nationally representative estimates of behavioral and clinical experiences of U.S. adults with diagnosed HIV infection. This analysis used Medical Monitoring Project data collected during 2015–2019 to examine ART adherence and reasons for missing ART doses among HIV-positive Hispanic/Latino MSM (1,673). On a three-item ART adherence scale with 100 being perfect adherence, 77.3% had a score of ≥85. Younger age, poverty, recent drug use, depression, and unmet needs for ancillary services were predictors of lower ART adherence. The most common reason for missing an ART dose was forgetting; 63.9% of persons who missed ≥1 dose reported more than one reason. Interventions that support ART adherence and access to ancillary services among Hispanic/Latino MSM might help improve clinical outcomes and reduce transmission.

The Medical Monitoring Project used a two-stage sampling method. During the first stage, 16 states and one territory were sampled from all U.S. states, the District of Columbia, and Puerto Rico. During the second stage, simple random samples of adults with diagnosed HIV infection were selected for each participating jurisdiction from the National HIV Surveillance System, a census of persons with diagnosed HIV infection in the United States. In-person or telephone interviews were conducted during the 2015–2018 data cycles,* in which self-reported sociodemographic characteristics, ART adherence, drug and alcohol use, and symptoms of depression^†^ and anxiety^§^ were ascertained.

ART adherence during the 30 days before the interview was assessed using a three-item scale; responses were aggregated and transformed into a previously validated composite score (range = 0–100), which has high internal reliability and is consistent with electronic drug monitoring measures (*4*,*5*). Ancillary services^¶^ were defined as services that enable and support participants’ retention in HIV care (*6*). Sustained viral suppression was defined as all viral load measurements in the previous 12 months documented as undetectable or <200 viral RNA copies/mL.** Reasons for most recent missed ART dose consisted of predefined options that respondents could select and were limited to the 2018 data cycle because of skip-pattern changes that limited comparability with earlier data cycles.

This analysis was limited to men who self-identified as Hispanic/Latino, regardless of race, who were currently taking ART and self-identified as MSM (i.e., gay or bisexual or who reported having had sex with one or more men during the previous 12 months) (1,673). Among HIV-positive Hispanic/Latino MSM, the three components of the ART adherence scale and the ART adherence scale score (dichotomized as ≥85 versus <85 on the basis of the distribution of scores), by selected characteristics, were examined by using weighted percentages with corresponding 95% confidence intervals (CIs). Characteristics associated with high ART adherence (score ≥85) were assessed by using a multivariable logistic regression model to describe adjusted prevalence ratios (aPRs) with predicted marginal means (*7*). Characteristics with bivariate associations with ART adherence (p<0.1) were eligible for possible inclusion in the model. Backward selection was used to determine final model selection, where eligible covariates with significant associations (p<0.05) were retained in the final model. Among persons who reported ever missing ≥1 ART dose (348 during the 2018 data cycle), reasons for most recent missed dose were described; participants could report more than one reason. Sustained viral suppression status was assessed, comparing those with higher adherence (i.e., adherence score ≥85) with those with lower adherence (i.e., adherence score <85), using a univariate prevalence ratio (PR). All analyses were weighted to adjust for individual nonresponse and poststratified to known population totals by age, race/ethnicity, and sex from the National HIV Surveillance System. Analyses were conducted using survey procedures in SAS software (version 9.4; SAS Institute) and SAS-callable SUDAAN (version 11.0.3; RTI International).

During 2015–2019, 57.4% of Hispanic/Latino MSM reported taking all ART doses during the previous month, 52.9% reported doing an excellent job taking their medications, and 69.2% reported always taking their medications as recommended (Table 1). ART adherence was high for 77.3% (Table 2). Younger persons and those at or below the federal poverty threshold were less likely to report high ART adherence. Reported ART adherence was lower among persons who reported drug use in the previous year (67.2%) than among those who did not (81.9%), among persons who reported a recent history of depression (66.3%) than among those who did not (79.9%), and among persons who had unmet needs for ancillary services (71.6%) than among those without unmet needs (83.0%). Anxiety and history of homelessness were not associated with ART adherence after adjustment for other factors. Among persons who had ever missed ≥1 ART dose, the most commonly reported reasons for the most recent missed dose were forgetting to take medication (63.1%), a change in daily routine or travel (42.3%), and having fallen asleep early or overslept (33.6%) (Table 3). Approximately 64% of persons who missed ≥1 dose reported multiple reasons for missing ART. Sustained viral suppression was more common among persons with ART adherence scores ≥85 (75.3%) than among persons with lower scores (59.7%; PR = 0.61; 95% CI = 0.51–0.74).

**TABLE 1:** Adherence to antiretroviral therapy (ART) among Hispanic/Latino men who have sex with men currently taking ART (N = 1,673) — Medical Monitoring Project, United States, 2015–2019

Interview question	No.*	% (95% CI)^†^
**How many days did you miss ≥1 dose of any of your HIV medicines?^§^**		
0	943	57.4 (54.7–60.1)
1–2	489	29.2 (26.7–31.7)
3–5	151	8.5 (6.9–10.1)
6–10	52	3.0 (1.9–4.0)
≥11	36	2.0 (1.3–2.6)
**How well did you do at taking your HIV medicines in the way you were supposed to?^§^**		
Very poor	18	0.9 (0.5–1.3)
Poor	21	1.3 (0.6–2.0)
Fair	86	5.5 (4.0–7.0)
Good	207	11.6 (9.9–13.3)
Very good	472	27.8 (25.1–30.5)
Excellent	869	52.9 (49.9–55.9)
**How often did you take your HIV medicines in the way you were supposed to?^§^**		
Never	14	0.7 (0.3–1.1)
Rarely	—^¶^	—^¶^
Sometimes	36	2.0 (1.4–2.7)
Usually	79	5.0 (3.7–6.4)
Almost always	374	22.7 (20.1–25.3)
Always	1,163	69.2 (66.4–72.0)

**Abbreviations:** CI = confidence interval; HIV = human immunodeficiency virus.

* Numbers might not sum to total because of missing data, and percentages might not sum to 100 because of rounding.

^†^ Percentages and corresponding CIs are weighted percentages.

^§^ Time frame for all questions is the 30 days before the interview.

^¶^ Value is excluded because coefficient of variation >0.30.

**TABLE 2:** Prevalence of medication adherence and association with selected sociodemographic characteristics among Hispanic/Latino men who have sex with men currently taking antiretroviral therapy (ART) (N = 1,673)— Medical Monitoring Project, United States, 2015–2019

Characteristic	Total no.*	Adherence score ≥85^†^		Unadjusted		Adjusted	
		No.*	% (95% CI)^§^	Prevalence ratio (95% CI)	P-value	Prevalence ratio (95% CI)	P-value
**Sociodemographic variables**							
**Age group (yrs)**							
18–29	192	126	65.7 (57.3–74.1)	0.79 (0.69–0.90)	<0.001	0.85 (0.76–0.96)	0.005
30–39	398	279	72.2 (66.9–77.5)	0.86 (0.79–0.94)		0.88 (0.80–0.96)	
40–49	482	372	77.8 (73.3–82.2)	0.93 (0.87–1.00)		0.94 (0.87–1.01)	
≥50	598	492	83.6 (80.4–86.7)	Reference		Reference	
**Education level**							
Less than high school	207	158	75.2 (68.3–82.2)	0.95 (0.86–1.05)	0.069	—^¶^	—
High school diploma or equivalent	342	242	72.6 (67.0–78.2)	0.92 (0.85–1.00)		—	—
More than high school	1,120	868	79.1 (76.3–81.8)	Reference		—	—
Household poverty level**							
Above threshold	1,024	801	79.7 (76.8–82.6)	Reference	0.002	Reference	0.024
At or below threshold	535	380	71.5 (66.8–76.2)	0.90 (0.83–0.96)		0.93 (0.87–0.99)	
**Homeless^††^**							
Yes	124	72	60.7 (50.9–70.5)	0.77 (0.66–0.91)	<0.001	—	—
No	1,546	1,197	78.5 (76.0–81.0)	Reference		—	—
**Risk behaviors**							
**Binge drinking, previous 30 days^§§^**							
Yes	405	289	74.0 (68.6–79.4)	0.94 (0.87–1.02)	0.142	—	—
No	1,254	974	78.4 (75.7–81.2)	Reference		—	—
**Drug use, previous 12 mos**							
Yes	548	349	67.2 (62.2–72.3)	0.82 (0.76–0.89)	<0.001	0.86 (0.80–0.93)	<0.001
No	1,114	916	81.9 (79.4–84.4)	Reference		Reference	
**Clinical variables**							
**Time since HIV diagnosis (yrs)**							
<5	377	279	72.3 (66.7–77.8)	0.91 (0.84–0.99)	0.058	—	—
5–9	386	290	77.4 (72.5–82.4)	0.98 (0.91–1.05)		—	—
≥10	905	698	79.2 (76.3–82.1)	Reference		—	—
**Symptoms of depression, previous 2 wks^¶¶^**							
Yes	323	206	66.3 (60.3–72.4)	0.83 (0.76–0.91)	<0.001	0.91 (0.83–1.00)	0.026
No	1,335	1,054	79.9 (77.3–82.5)	Reference		Reference	
Symptoms of generalized anxiety disorder, previous 2 wks***							
Yes	349	234	69.5 (63.2–75.8)	0.88 (0.80–0.96)	0.001	—	—
No	1,315	1,031	79.4 (76.9–81.9)	Reference		—	—
**Attended Ryan White–funded facility for usual care**							
Yes	1,165	878	76.5 (73.5–79.5)	0.96 (0.90–1.03)	0.266	—	—
No	436	341	79.7 (75.1–84.3)	Reference		—	—
**Retained in care, previous 12 mos^†††^**							
Yes	1,462	1,122	78.2 (75.8–80.7)	Reference	0.180	—	—
No	153	108	72.2 (63.1–81.3)	0.92 (0.81–1.05)		—	—
**Health insurance type**							
Any private insurance	652	519	80.3 (76.7–83.9)	Reference	0.130	—	—
Public insurance only	764	557	75.4 (71.7–79.1)	0.94 (0.88–1.00)		—	—
Uninsured or Ryan White HIV/AIDS Program coverage only	239	181	75.0 (68.8–81.2)	0.93 (0.85–1.03)		—	—
**Had at least one unmet need for ancillary services, previous 12 mos^§§§^**							
At least one unmet need	832	581	71.6 (67.7–75.5)	0.86 (0.81–0.92)	<0.001	0.89 (0.83–0.95)	0.001
Received or did not need services	836	687	83.0 (80.0–85.9)	Reference		Reference	
**Received adherence support services**							
Yes	624	474	77.7 (73.8–81.5)	Reference	0.851	—	—
No	1,042	794	77.2 (74.2–80.2)	0.99 (0.94–1.06)		—	—

**Abbreviations:** CI = confidence interval; HIV = human immunodeficiency virus.

* Numbers are unweighted.

^†^ Adherence scale score is composite of three variables (number of missed days of ART, how often respondent took ART correctly, and how good of a job taking ART respondent reported) and ranges from 0 to 100, with 100 indicating perfect adherence.

^§^ Percentages and corresponding CIs are weighted percentages.

^¶^ Dash indicates that a value is not applicable.

** Poverty guidelines as defined by the U.S. Department of Health and Human Services.

^††^ Living on the street, in a shelter, in a single-room–occupancy hotel, or in a car.

^§§^ Binge drinking for men is defined as five or more alcoholic drinks in one sitting.

^¶¶^ Depression was assessed by using the eight-item Patient Health Questionnaire algorithm.

*** Anxiety was assessed by using the Generalized Anxiety Disorder Scale.

^†††^ Retention in care was defined as documentation of two indications of outpatient HIV care, including a documented visit with an HIV provider, a documented CD4^+^ or viral load test, or a documented resistance test or tropism assay, ≥90 days apart during the previous 12 months.

**^§§§^** Ancillary services include HIV case management services, adherence counseling services, AIDS Drug Assistance Program services, patient navigation services, HIV peer group support, dental services, drug or alcohol counseling or treatment, mental health services, transportation assistance, shelter/housing services, Supplemental Security Income, Social Security Disability Insurance, food assistance, meals or food services, interpreter services, or legal services.

**TABLE 3:** Reason for most recent missed antiretroviral therapy (ART) dose* among Hispanic/Latino men who have sex with men (MSM) with diagnosed human immunodeficiency virus (HIV) infection currently taking ART and number who reported multiple reasons — Medical Monitoring Project, United States, 2018–2019^†^

Reason	No.^§^	% (95% CI)^¶^
**Forgetting to take HIV medicines**		
Yes	222	63.1 (55.7–70.5)
No	126	36.9 (29.5–44.3)
**Change in daily routine or travel**		
Yes	156	42.3 (36.8–47.9)
No	192	57.7 (52.1–63.2)
**Fell asleep early or overslept**		
Yes	121	33.6 (28.7–38.5)
No	227	66.4 (61.5–71.3)
**Problem getting prescription or refill for HIV medicines**		
Yes	63	18.8 (14.5–23.0)
No	285	81.2 (77.0–85.5)
**Felt depressed or overwhelmed**		
Yes	67	17.6 (13.7–21.6)
No	281	82.4 (78.4–86.3)
**Did not feel like taking HIV medicines**		
Yes	41	13.0 (8.9–17.1)
No	307	87.0 (82.9–91.1)
**Drug or alcohol use**		
Yes	41	11.8 (7.9–15.7)
No	307	88.2 (84.3–92.1)
**Side effects from HIV medicines**		
Yes	38	10.7 (7.4–14.0)
No	309	89.3 (86.0–92.6)
**Problem paying for HIV medicines**		
Yes	25	6.6 (3.4–9.8)
No	323	93.4 (90.2–96.6)
**In the hospital or too sick to take HIV medicines**		
Yes	16	4.5 (2.6–6.5)
No	332	95.5 (93.5–97.4)
**Reported multiple reasons for missing ART**		
Yes	223	63.9 (58.1–69.7)
No	125	36.1 (30.3–41.9)

**Abbreviation:** CI = confidence interval.

* Respondents could select multiple reasons for missing a dose.

^†^ Data limited to 2018 cycle because of changes in skip-pattern preceding questions.

^§^ Numbers might not sum to total because of missing data, and percentages might not sum to 100 because of rounding.

^¶^ Percentages and corresponding CIs are weighted percentages.

## Discussion

Although high overall, self-reported ART adherence among HIV-positive Hispanic/Latino MSM was lower among younger persons, those living at or below poverty, and those who reported drug use, depression, and unmet needs for ancillary services. The most commonly reported reason for last missed ART dose was forgetting to take it; three in five persons reported multiple reasons. These results indicate possible avenues for interventions to help Hispanic/Latino MSM engage in care and remain ART-adherent.

Ancillary services (e.g., counseling for mental health and substance use disorders, financial support, and assistance with food and housing) might reduce barriers to ART adherence. Colocating these services with outpatient care (e.g., HIV patient-centered medical home model of the Ryan White HIV/AIDS Program)(*8*) can encourage engagement and retention in HIV care. In a study of ART adherence among African American and Hispanic/Latino MSM, younger participants reported better adherence when their care location also offered ancillary services to help them address other needs (*3*). Making these services more broadly available and easily accessible might remove barriers to ART adherence and improve health outcomes.

Approximately two thirds of persons who had missed ≥1 ART dose reported having forgotten to take it. Interventions that include reminders might help prevent these lapses. An analysis of systematic reviews of ART adherence interventions found that text messaging interventions were among the most successful for improving both self-reported adherence and viral load (*9*). Other interventions that have increased ART adherence include peer support and cognitive behavioral therapy. Interventions that include multiple strategies were more likely to increase ART adherence, although effects were often short-lived (*10*).

In 2019, the U.S. Department of Health and Human Services proposed Ending the HIV Epidemic: A Plan for America (EHE) (*2*). Two of the four primary pillars of EHE are early HIV diagnosis and treatment of HIV infection to help persons achieve and maintain viral suppression to prevent morbidity and further transmission. CDC is working with state and local partners and other stakeholders to use interventions that support the four EHE pillars.^††^ For example, Sin Buscar Excusas/No Excuses is a video-based intervention for Hispanic/Latino MSM that is intended to prevent transmission by increasing sexual safety, HIV testing, and HIV care.^§§^ Another intervention, Helping Enhance Adherence to Antiretroviral Therapy (HEART), helps patients develop individualized adherence plans by using problem-solving activities to identify and address their ART adherence barriers. HEART also incorporates a patient-identified support partner to aid in meeting ART adherence goals.^¶¶^

The findings in this report are subject to at least three limitations. First, data ascertained through participant interviews, including information on ART adherence, were based on self-report and might be subject to social desirability or recall bias. Second, results were adjusted to minimize nonresponse bias on the basis of standard methodology; however, the possibility of residual nonresponse bias remains. Finally, reasons for missing ART doses might not be exhaustive.

This report highlights barriers to ART adherence faced by Hispanic/Latino MSM with diagnosed HIV infection. Culturally tailored interventions aimed at improving adherence, particularly among Hispanic/Latino MSM who are younger, live in poverty, use drugs, and have unmet needs for ancillary services, might improve viral suppression, leading to better health outcomes and decreasing HIV transmission.

**Summary**

**What is already known about this topic?**

Antiretroviral therapy (ART) adherence is crucial for viral suppression, a critical outcome for maintaining health in persons with HIV infection. Hispanic/Latino men who have sex with men (MSM) have disproportionately high HIV infection rates; their barriers to ART adherence have not been extensively explored.

**What is added by this report?**

ART adherence was lower among younger Hispanic/Latino MSM and those who experienced poverty or reported drug use, depression, or unmet ancillary service needs. The most common reason for missing ART doses was forgetting (63.1%); 63.9% who missed doses reported multiple reasons.

**What are the implications for public health practice?**

Expanding access to ancillary services among Hispanic/Latino MSM, particularly those experiencing barriers to ART adherence, might improve clinical outcomes.

## Footnotes

* Interviews are conducted during June–May for each annual cycle; this analysis combined 2015–2018 cycles. Response rates for participants ranged from 40% to 46% by cycle year.

† Symptoms of depression during the previous 2 weeks were assessed using the eight-item Patient Health Questionnaire.

§ Symptoms of anxiety during the previous 2 weeks were assessed using the Generalized Anxiety Disorder Scale.

¶ Ancillary services include HIV case management services, ART adherence counseling services, AIDS Drug Assistance Program services, patient navigation services, HIV peer-group support, dental services, drug or alcohol counseling or treatment, mental health services, transportation assistance, shelter/housing services, Supplemental Security Income, Social Security Disability Insurance, food assistance, meals or food services, interpreter services, or legal services.

** Persons with unknown viral load measurements were analyzed with those with detectable viral loads.

†† https://www.cdc.gov/hiv/effective-interventions/a-to-z.html.

§§ https://www.cdc.gov/hiv/effective-interventions/prevent/sin-buscar-excusas.

¶¶ https://www.cdc.gov/hiv/effective-interventions/treat/heart.
